# Involvement of Stat1 in the Phagocytosis of *M. avium*

**DOI:** 10.1155/2012/652683

**Published:** 2012-06-28

**Authors:** Sabrina Dominici, Giuditta Fiorella Schiavano, Mauro Magnani, Costantina Buondelmonte, Angela Gabriela Celeste, Giorgio Brandi

**Affiliations:** ^1^Section of Biochemistry and Molecular Biology, Department of Biomolecular Sciences, University of Urbino “Carlo Bo”, Via Saffi No. 2, 61029 Urbino, Italy; ^2^Section of Toxicological Hygienic and Environmental Sciences, Department of Biomolecular Sciences, University of Urbino “Carlo Bo”, Via Saffi No. 2, 61029 Urbino, Italy

## Abstract

*Mycobacterium avium* is an intracellular pathogen preferentially infecting human macrophages where they activate the JAK/STAT1 pathway. This activation enhances the survival of infected cells, but, at the same time, makes macrophages optimal targets for drugs development against p-tyr_701_stat1. In this study, we demonstrate that the fast and transient activity of the JAK/STAT1 pathway occurs immediately after macrophages internalization of heat-killed *M. avium* or inert particles. Furthermore, we show that a persistent Stat1 pathway activation occurs only when an intracellular *M. avium* infection is established in macrophages. These results strongly indicate different mechanisms of p-tyr_701_Stat1 activation. In particular, here we report findings aiming at explaining the short-time enhancement of p-tyr_701_Stat1 and shows its predominant relationship with Fc**γ**Rs engagement during the internalization process. Furthermore, we demonstrate that opsonized live *M. avium* is phagocytosed by macrophages involving membrane receptors not related with JAK/STAT1 signalling pathway. On the contrary, heat-inactivated bacilli or latex particles seem to be internalized only after involvement of Fc**γ**Rs and subsequent Stat1 phosphorylation.

## 1. Introduction

*Mycobacterium avium* is an opportunistic bacterial pathogen that causes serious infection in immunocompromised individuals, such as in human immunodeficiency virus-positive patients [1]. Macrophages are a common target for mycobacterial infections that benefit from avoiding contact with the immune system. It is known that within the macrophages, *M. avium* persists and replicates in a large percentage of immunocompromised patients, and hosts' response against this bacillus is ineffective in eradicating mycobacteria from phagocytic cells [2]. When macrophages make contact with bacteria, several signal-transduction pathways are activated [3]. In a previous study, we demonstrated that the *M. avium* infection induces the expression and phosphorylation of signal transducer and activator of transcription type 1 (Stat1), a protein involved in many cell functions. This makes infected macrophages as optimal targets for drugs active against p-tyrStat1 [4]. Recent findings suggest an important involvement of Stat proteins in bacterial infections, and these hypotheses lead to innate immune responses; in fact, the JAK/STAT1 pathway constitutes one of the main ways to activate macrophages, upregulating the expression of many different genes associated with the secondary cell responses leading to macrophage activation or apoptosis [5]. These mechanisms have probably developed to defend the host against bacteria and other pathogens [6]. Although, in general, Stat1 pathway activation has always been correlated with INF-*γ*, recent findings regarding macrophages exposed to mycobacterial infection propose new mechanisms where Stat1 phosphorylation are independent of INF-*γ* [7–15]. In our previous paper, we postulated the hypothesis that p-tyr_701_Stat1 overexpression after *M. Avium*-infected macrophage depends on the establishment of infection and does not seem to be correlated with the production of INF-*γ* or -*α* [16]. We also demonstrated that the permanent phosphorylation of Stat1 is important for macrophage survival after establishment of *M. avium* infection, ensuring the optimal condition for bacteria viability [4–17]. 

Previously (data not shown), we found the p-tyr_701_Stat1 overexpression after short times (0–48 hours) in *M. avium* infected cells as well as in macrophages-internalizing heat-killed bacteria or latex beads. After 48 hours, infected cells maintained the p-tyr_701_Stat1 expression levels at high values, on the contrary after the heat-killed *M. avium* and latex particles phagocytosis, the protein expression back permanently to the control value. This suggests an involvement of the JAK/STAT1 pathway closely related to the act of phagocytosis, presumably involving specific membrane receptors. The phagocytosis of microbes involves a broad spectrum of receptors that participate in particle recognition and internalization. Some of these receptors are capable of transmitting intracellular signals that trigger phagocytosis, while other receptors appear to participate primarily in binding to increase the efficiency of internalization [18–22]. In “*in vitro*” methodologies, live or inactivated bacteria, or latex particles are usually exposed to pooled human serum to facilitate their uptake into the phagocytic cells. Complement-opsonized particles are recognized and internalized via specific complement receptors (CRs). Complement proteins present in the serum can opsonize microbes for phagocytosis by the C3b or C3bi receptors on macrophages; Several receptor that participate in phagocytosis of complement-opsonized particles, including CR1, CR3, and CR4 are expressed on macrophages [23, 24]. CR1 binds C3b, C4b, C3bi, and is thought to participate mainly in particles binding [25]. CR3 and CR4 are integrin family members made up of heterodimers of different *α* chains and a shared *β* chain [23]. These two receptor bind specifically to C3bi and are responsible for particles internalization. It was hypothesized that pathogenic mycobacteria recruit the complement fragment C2a to form a C3 convertase and generate opsonically active C3b for penetrating into macrophages [26, 27]. Phagocytes such as macrophages or neutrophils express also different combinations of Fc*γ*Receptors (Fc*γ*R), able to recognize IgG-opsonized particles. Macrophages expressing different combinations of Fc*γ*Rs fall into the two different typologies, activating receptors that bind opsonized particles, and inhibitory Fc*γ*Rs that block phosphoinositide signalling [28, 29]. Several authors indicate a correlation between Fc*γ*Rs engagement and the outbreak of inflammatory response. Through these cellular responses, Fc*γ*Rs mediate the ingestion of viruses, bacteria, and parasites, as well as the antibody-dependent killing of cells expressing viral or tumor antigens [17, 30–32]. It has been recently demonstrated that rapid JAK/STAT1 signalling pathway activation may be related to F*γ*Rs engagement. The described mechanisms are closely related to phagocytic activity and indicate that the JAK/STAT1 activation after Fc*γ*Rs engagement, lies in the release of cytokines, like IFNs, resulting from inflammatory responses [33]. The focus of this work is to try to clarify the different mechanisms of *M. avium* internalization by identifying the membrane receptors mediating phagocytosis and their relation with JAK/STAT1 pathway activity. The work highlights the difference between viable *M. avium* and heat-killed *M. avium *internalization mechanisms and also correlates JAK/STAT1 activation through Fc*γ*R engagement. This paper provides researchers with useful information to clarify the internalization mechanisms of mycobacterial infections related to cellular activation.

## 2. Materials and Methods

### 2.1. Mycobacteria

*M. avium *strain 662, used in this study, were obtained from the blood of patients with AIDS who were admitted to “L. Sacco”, Hospital (Milan, Italy) and who had never been treated for mycobacterial infection. Bacteria were routinely grown in Middlebrook 7H10 Agar supplemented with oleic-albumin-dextrose catalase (OADC) enrichment (Difco Laboratories, Detroit, USA). Bacterial cell suspensions were obtained culturing smooth transparent colonies in Middlebrook 7H9 broth supplemented with albumin-dextrose catalase (ADC) enrichment (Difco Laboratories, Detroit, USA), at 35°C for 7–10 days (logarithmic phase). Bacterial cells were harvested and washed in Hank's balanced salt solution (HBSS). One aliquot was taken up and heat treated (67°C for 15 min) to kill bacilli. The complete heat-inactivation of bacteria was evaluated by spreading 100 *μ*L of the bacterial suspension on Middlebrook 7H10 Agar.

All suspensions before each use were sonicated for 6 s (power output, 2.5 W/s) to disperse clumps of bacilli and its turbidity was adjusted to an optical density of 0.12 (at 650 nm), corresponding to approximately 1 × 10^8^ bacilli per mL.

### 2.2. Monocyte-Derived Macrophages 

Monocyte-derived macrophages were prepared from leukocyte buffy coats obtained from healthy donors and purified as previously described [34]. Briefly, the peripheral blood mononuclear cells were isolated by Histopaque 1077 (Sigma Chemical Co.) gradient centrifugation and monocytes were separated from lymphocytes by adherence to plastic dishes, scraped, washed twice and suspended in RPMI 1640 medium (cell culture tested) with 10% (v/v) inactivated fetal bovine serum and 1% antibiotics (v/v). Aliquots of 1 × 10^6^ cells were seeded in 35-mm Petri dishes and cultured at 37°C in 5% CO_2_ atmosphere for 10 days, at which time the monocytes had matured into macrophages.

### 2.3. Opsonization and Phagocytosis

 Live and dead bacteria or 1 *μ*m latex beads-FITC conjugated (Sigma Aldrich) were suspended in RPMI 1640 medium supplemented with 10% of human pooled serum (AB), heat de-complement-human pooled serum (d-AB), and human pooled serum ultra-filtrate with 10,000 cut off membrane (u-AB) and with 8% (v/v) human affinity purified Immunoglobulins (IgG) to promote opsonization and successively internalization into phagocytic cells. Bacteria or fluorescent latex beads (Sigma Aldrich) were then added to mature macrophages at a ratio of 60 : 1 (bacilli or beads/macrophage) and incubated for 4 hours at 37°C in 5% CO_2_. In a set of experiments, viable and heat-killed bacteria were added in an equivalent ratio and incubated in the same way in presence of 2 *μ*g/mL of monoclonal antibody mAb IV.3 blocking Fc*γ*RIIA receptor inhibitor (kindly provided by Professor Mauro Torti, Department of Biochemistry, University of Pavia) [35]. After incubation, unbound or unphagocytosed bacteria or fluorescent latex beads were removed, respectively, by extensive washing with HBSS. Macrophages exposed to live bacteria were lysed to assess the number of intracellular colony-forming units by dilution plate counts on Middlebrook 7H10 Agar. Internalized bacteria or latex beads were Ziehl-Nelsen stained and observed in light or fluorescence microscopy to assess the number of phagocytosed bacilli and latex particles, respectively. The number of internalized materials in all experimental conditions, was evaluated in at least two independent experiments.

### 2.4. Western Blot Assay for Stat1 and p-tyr_**701**_Stat1 Evaluation

At the experimental times 0, 24, 48, and 72 hours after exposure to bacteria or latex particles, macrophages were washed twice with phosphate buffered saline pH 7.4 (PBS) and successively lysed in lyses buffer. The lyses buffer consists of 50 mM Tris-HCl pH7.8, 2% (w/v) SDS (sodium dodecyl sulfate), 5 mM EDTA (ethylenediaminetetraacetic acid), 109 mM NEM (N-ethylmaleimide), protease inhibitors (2 mg/mL leupeptin) (2 mg/mL pepstatin) and 4 mM AEBSF (4-(2-Aminoethyl) benzenesulphonyl fluoride) and 1 mM PMSF (Phenyl-methyl sulphonyl fluoride), and phosphatase inhibitors ( 1 mM sodium orthovanadate and 1 mM of sodium fluoride). Whole cell lysates were boiled immediately for 5 min and centrifuged at 6,000 ×*g* for 13 min. The protein concentration of cell extracts was determined by the Lowry assay [36]. For the detection of Stat1 and p-tyr_701_Stat1, ten micrograms of cell extracts were resolved on 7.5% SDS-PAGE and then blotted on Hybond-C Extra nitrocellulose membrane (Amersham Pharmacia Biotech, Italy) for 60 minutes at 100 V with a Bio-Rad trans-blot (Bio-Rad laboratory, Germany) [37, 38]. For the immunoassay, membranes were treated with blocking solution (5% (w/v) nonfat dry milk dissolved in TBS (150 mM NaCl, 50 mM Tris pH 7.5)) and maintained for 1 hour at room temperature. The specific immunecomplexes were revealed after incubation with three different polyclonal antibodies: anti-p-tyr_701_Stat1 (Cell Signalling), anti-Stat1 (Santa Cruz Biotechnology, Inc.), and anti-actin (Sigma Aldrich). The immune reactive bands were revealed after successive exposure to a horseradish peroxidase-conjugated anti-rabbit IgG (Bio-Rad laboratory, Germany) and followed by enhanced chemiluminescence reaction (ECL, Amersham Pharmacia Biotec, Italy) [39]. Quantitative analysis was performed by a ChemiDoc system and Quantity One Program system (BioRad laboratory). Statistical analyses were performed with GraphPad Prism 4 software.

## 3. Results and Discussion

### 3.1. Stat1/p-tyr_**701**_Stat1 Pathway, Activation at Short Time

**Figure 1:**
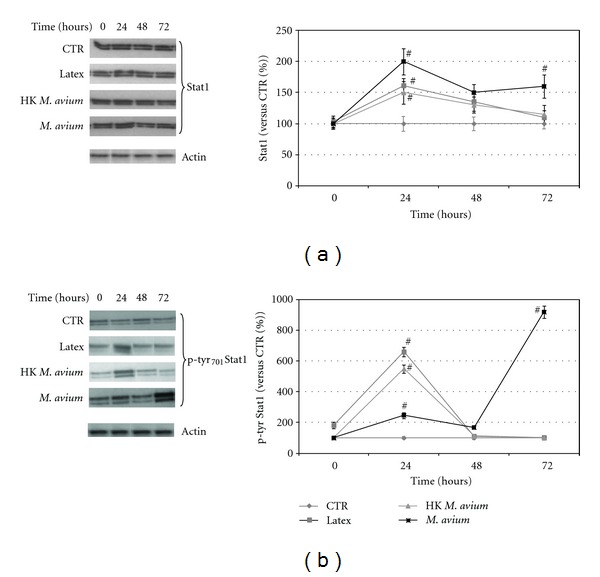
Time course of JAK/STAT1 signalling pathway activation after internalization of latex particles, heat-killed and live *M. avium* into macrophages. (a) Left: figure of western blot analysis of Stat1 and actin levels at 0, 24, 48, and 72 hours after uptake. The control (CTR) is represented by untreated macrophages; right: in the graph, we report the quantitative analysis of Stat1 normalized to the amount of actin and expressed as percentage of control value which is placed as 100%. (b) Left: figure of western blot analysis of p-tyr_701_Stat1 and actin levels at 0, 24, 48, and 72 hours after uptake. The control (CTR) is represented by untreated macrophages; right: in the graph, we report the quantitative analysis of p-tyr_701_Stat1 normalized to the amount of actin and expressed as percentage of control value which is placed as 100%. The results are the mean of five different experiments. Statistical analyses were performed with the Student's *t*-test (Prism 4 Graph Pad software): (#) significant difference from control (CTR) *P* < 0.05.

In our previous work, we noted that live *M. avium* infection in macrophages maintained JAK/STAT1 pathway active for a long time (for more than 7 days), consequent to the establishment of intracellular infection and bacteria replication in the phagosome [4]. The persistent major expression of p-tyr_701_Stat1 in infected macrophages proposed experimental approaches aimed to selectively eliminate persistently infected cells, with the drugs active against p-tyr_701_Stat1. The increased expression for a long time (more than 48–72 hours) does not occurr after macrophages internalization of heat-inactivated *M. avium* or latex beads, the two conditions used as controls [4]. In fact, during these experiments, we have revealed a very evident increment in the Stat1 and in p-tyr_701_Stat1 proteins' expression levels after a short time (we considered the interval time 0–48 hours), after macrophages exposition to live and heat-inactivated *M. avium* and latex particles. On the basis of these results, we evaluated the involvement of the Stat1 signalling pathway to internalization events in human macrophages. In Figures 1(a) and 1(b), we show the time course of activation/deactivation of JAK/STAT1 pathway in the various experimental conditions. Western blot analysis of Stat1 and p-tyr_701_Stat1 showed that the proteins expression reached maximal values at 24 hours after cellular uptake in all conditions assessed. At this time point, in the case of viable infection (live *M. avium*), the Stat1 and p-tyr_701_Stat1 expression levels were 200% and 250% increased as compared to control macrophages, respectively (Figures 1(a) and 1(b)).At 48 hours after live *M. avium* uptake, the Stat1 and p-tyr_701_Stat1 expression levels were reduced to 150% and 180% as compared to the CTR, respectively, but after 48 hours protein levels increased steadily for a long time (for more than 72 hours) (Figures 1(a) and 1(b)), according to the previous data [4]. In macrophages internalizing heat-killed bacteria or latex particles, the increment of Stat1 and p-tyr_701_Stat1 after 24 hours was also very evident. In fact, at 24 hours after the heat-killed bacteria internalization the Stat1 and p-tyr_701_Stat1 levels were approximately 150% and 550% in respect to CTR, respectively. At 48 hours after cellular uptake, the protein levels back to normal values. In macrophages internalizing latex particles, Stat1 and p-tyr_701_Stat1 expression levels reached values up to 160% and 650%, respect to CTR, respectively, and at 48 hours after cellular uptake, the protein levels took the value of control. In heat-killed bacteria and latex beats internalization contrary to viable infection, no further changes in protein levels were observed at later time point (72 hours) (Figures 1(a) and 1(b)). This led us to postulate the existence of two different mechanisms of internalization for live infections and inactivated bacteria. These findings suggest a potential engagement of certain membrane receptors correlated with JAK/STAT1 signalling pathway activation and able to mediate bacilli phagocytosis or particles internalization in macrophages. For this reason, a research aimed to correlate the membrane receptors, Stat1 activation and internalization process into macrophages was performed. It is known that lipopolysaccharide (LPS) molecules activate JAK/STAT1 pathway through LPS-induced CD40 expression or through unknown mechanisms [40]. To demonstrate that the short time expression of p-tyr_701_Stat1 was independent of bacterial endotoxins, all experiments were performed in pyrogen-free conditions, choosing as further control pyrogen-free latex particles. As shown in Figures 1(a) and 1(b), all the experiments were performed using heat-inactivated bacteria in parallel with latex particles. The Stat1 and p-tyr_701_Stat1 reaching the same expression levels at each point of the time course (0–24–48–72 hours). These results demonstrate that the overexpression of p-tyr_701_Stat1 after short times (0–48 hours) does not depend on pyrogenic contaminants but rather from the mechanism of internalization activating JAK/STAT1 signalling pathway.

### 3.2. Fc*γ*R and JAK/STAT1 Signalling Pathway Activity 

**Figure 2:**
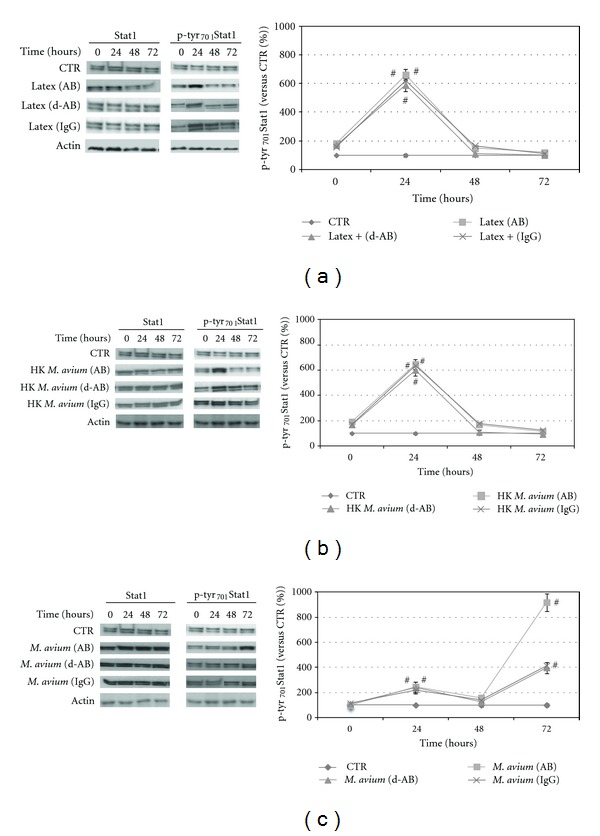
Time course of Stat1/p-tyr_701_Stat1 signalling pathway activation after internalization of live *M. avium* (a), heat-killed (H.K.) *M. avium* (b), and latex particles (c), in the different opsonization conditions. The opsonization methods are as follow: human pooled serum (AB), decomplemented human serum (d-AB), and purified human IgG (IgG). (a) Left: figure of western blot of p-tyr_701_Stat1 and actin levels at 0, 24, 48, and 72 hours after uptake of live *M. avium*; right: in graph, we report the time-dependent expression of p-tyr_701_Stat1. The protein expression levels were normalized to the amount of actin and expressed as percentage of control value which was placed as 100%. (b) Left: figure of western blot of p-tyr_701_Stat1 and actin levels at 0, 24, 48, and 72 hours after phagocytosis of heat-inactivated *M. avium*; right: in the graph, we report the time-dependent expression of p-tyr_701_Stat1. The protein expression levels were normalized to the amount of actin and expressed as percentage of control value which was placed as 100%. (c) Left: figure of western blot of p-tyr_701_Stat1 and actin levels at 0, 24, 48, and 72 hours after internalization of latex particles; right: in the graph, we report the time-dependent expression of p-tyr_701_Stat1. The protein levels were normalized to the amount of actin and expressed as percentage of control value which was placed as 100%. The results are the mean of three different experiments. Statistical analyses were performed with Student's *t*-test (Prism 4 Graph Pad software). (#) significant difference from control (CTR) *P* < 0.05.

We performed experiments aimed to clarify which of the classical membrane receptors could be directly related to the short time of p-tyr_701_Stat1 overexpression after cell contact with live or inactivated bacteria or latex particles. In our experimental conditions, live or heat-inactivated *M. avium* and latex particles as further control were exposed to pooled human serum (AB) in order to promote their phagocytosis through opsonization [41]. To investigate the membrane receptor/s related to JAK/STAT1 pathway activity, we performed different opsonization procedures. In Figures 2(a), 2(b), and 2(c), we show short-time Stat1/p-tyr_701_Stat1 expression levels in the macrophages internalizing live (Figure 2(a)) or heat-inactivated bacteria (Figure 2(b)) or latex particles (Figure 2(c)) after their exposure to human pooled serum (AB) decomplemented human pooled serum (d-AB), and affinity purified human IgG (IgG). In all experimental conditions, the results with serum (AB), also evidenced strong p-tyr_701_Stat1 expression level after 24 hours (600% in the heat-killed *M. avium* and latex particles and 200% in live *M. avium* conditions). It is known that the increment of the protein Stat1 and the presence of the phosphorylated form p-tyr_701_Stat1 indicates the activation of the JAK/STAT1 pathway [42]. At this time point (24 hours) the internalization entity was also evaluated as number of bacteria or latex particles for cell. The results show that the mean number of materials were up to 8.0 and 15.9 bacteria/cell in the case of live and heat-killed *M. avium,* respectively, and 5.4 beats/cell in the case of latex particles. We hypothesized that in the case of serum AB opsonization, the strong expression of p-tyr_701_Stat1 in all experimental conditions should be correlated both with the complement receptors (CRs) that with the Fc*γ* receptor engagement. To investigate membrane receptor directly involved with JAK/STAT1 pathway activation, excluding complement component, we opsonized bacteria and latex particles with serum d-AB and with purified human IgG (Figures 2(a), 2(b), and 2(c)). Also in these cases, the p-tyr_701_Stat1 expression levels reach values up to about 600% (CTR set as 100%) in the heat-killed *M. avium* and latex particles and 200% in live *M. avium*. Also in these experimental conditions, the internalized number of bacteria or latex beats was counted. The mean number of materials internalized after d-Ab opsonization were up to 2.1 and 16.3 bacteria/cell in the case of live and heat-killed *M. avium,* respectively, and 7.7 beats for cell in the case of latex particles. After human IgG opsonization, the phagocytosis entity was 5.0 and 15.0 bacteria for cell in live and heat-killed *M. avium,* respectively, and the latex particles internalized were 9.3 beats/cell. These results demonstrate an important increment in p-tyr_701_Stat1 expression levels close to internalization events in human macrophages, involving the Fc*γ*Rs. This mechanism, which is particularly evident at 24 hours after uptake, is presumably related to innate immune response as a preventive defense mechanism [6]. As described above, in *M. avium-*infected cells, the p-tyr_701_Stat1 expression value was three times less than that shown after heat-inactivated bacteria and latex particles internalization (Figures 2(a), 2(b), and 2(c)). These findings would indicate a further internalization mechanism, in the case of live *M. avium* engaging, in addition to Fc*γ*R, other membrane receptors working in synergy with Fc*γ*Rs. As reported by various authors, live *M. avium* enter into macrophage via C3b complement receptor [26, 27] that presumably not involving JAK/STAT1 signalling pathway, as demonstrated by the results obtained after bacteria opsonization with d-Ab or IgG (Figures 2(a), 2(b), and 2(c)). Furthermore, in the experimental conditions represented by live and heat-killed *M. avium* and latex particles, the number of internalized materials was variable depending by opsonization procedures. However, the p-tyr_701_Stat1 expression levels were totally independent from internalization entity, and this represent another important aspect to highlight. For reinforcing this hypothesis, we have opsonized latex beads with ultra filtered human pooled serum (u-AB). In this experiment, the uptake of particles resulted very high so as to make their enumeration difficult; however, the Stat1 and p-tyr_701_Stat1 expression levels remained at the control values (data not shown). This confirmed that the JAK/STAT1 pathway activity was not significantly influenced by the materials internalized into phagocytic cells. These findings indicate that the peak of expression at brief time of p-tyr_701_Stat1 was related to the membrane receptor involved in the phagocytic event and related to JAK/STAT1 activity and is not correlated with the number of internalizing materials.

### 3.3. Correlation between Internalization Entity and p-tyr_701_Stat1 Expression

**Figure 3:**
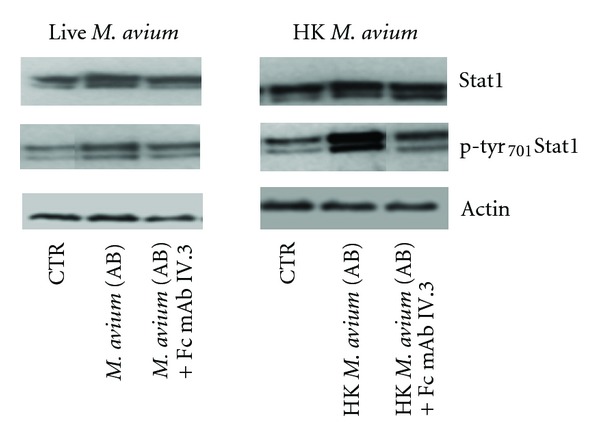
we show the western blotting figure of Stat1 and p-tyr_701_Stat1 expression levels after internalization by macrophages of the live and heat-killed *M. avium* opsonized with the serum AB, in presence and absence of Fc*γ*R inhibitor (mAb Fc IV.3).

 In Figure 3 and Table 1, we show the results derived from a set of experiments aimed to correlate Fc*γ*R engagement, JAK/STAT1 signalling pathway activity, and live or heat-inactivated bacteria internalization by phagocytic cells. For these experiments, macrophages were exposed to live or heat-killed *M. avium* opsonized either with serum AB in presence or absence of monoclonal antibody mAb IV.3 blocking Fc*γ*RIIA receptor. In fact, it is known that Fc*γ*RIIA mediates the phagocytosis of Ag-Ab complexes in macrophages and contains, in its cytoplasmatic tail, tyrosines that become phosphorylated upon receptor aggregation. Furthermore, Fc*γ*RIIA mediates efficient binding and phagocytosis of opsonized bacteria and to trigger tyrosine phosphorylation of several proteins [43–45]. Stat1/p-tyr_701_Stat1 expression levels evaluation and bacterial internalization were detected at the peak of expression of these proteins (24 hours after cell exposition). As shown in Figure 3 and Table 1, live *M. avium* internalization into macrophages induces a great expression of Stat1 and p-tyr_701_Stat1 up to 200% and 247%, respectively (compared to CTR cells placed as 100%), and is associated with an internalization activity reaching value up to 12.2 ± 3.3 bacteria/cell. The phagocytic activity also persists after Fc*γ*RIIA inhibition (11.3 ± 2.2 bacteria/cell; 95% versus live *M. avium* opsonized with serum AB set as 100%), even if Stat1/p-tyr_701_Stat1 expression levels return to control. These data have been compared to those obtained after heat-killed bacteria phagocytosis into macrophages in which case Stat1 and particularly p-tyr_701_Stat1 expression levels increased strongly (148% and 658%, respectively, as compared to CTR) and back to control values after Fc*γ*RIIA inhibition. Contrary to viable *M. avium*, the number of heat-inactivated bacteria ingested by macrophages decreased from 13.2 ± 3.5 to 5.0 ± 2.0 bacteria per cell (as 35% versus heat-inactivated *M. avium* opsonized with serum AB). These findings, furthermore, demonstrated the correlation between *M. avium* internalization through engagement of Fc*γ*R and Stat1 pathway activity at short times; this seems to be a preventive defense mechanism put in place by macrophages [6]. Furthermore, the results show different internalization mechanisms between live and heat-killed bacteria. In the case of *M. avium*, we postulated the mechanism of phagocytosis involving membrane receptors, as complement receptors (CRs) cooperating in phagocytic event with Fc*γ*R. In our experimental model, the CRs engagement by live *M. avium* not involve the activity of the JAK/STAT1 signalling pathway at short time (0–48 hours) [46]. In fact, the C3b-mediated live *M. avium* internalization, promoting the role of macrophages as bacterial reservoir, and for this reason Stat1 and p-tyr_701_Stat1 were strongly expressed for long time (starting to after 48 hours after infection) [4]. On the contrary, in the heat-killed *M. avium* internalization, we postulate a direct engagement of the only Fc*γ*R which is closely related to JAK/STAT1 pathway activation at short times (0–48 hours). The same results were also confirmed after addition of JAK1 inhibitor added in the different experimental conditions. In a further study, we will investigate the short-time expression of p-tyr_701_Stat1 after engagement of Fc*γ*Rs notably correlated with inflammatory response and cytokine release [33]. The internalization entity of live or dead bacteria was also correlated with Stat1 and p-tyr_701_Stat1 expression levels. As shown in Figure 3 and Table 1, the number of bacteria per cells was independent from the Stat1/p-tyr_701_Stat1 expression levels.

## 4. Conclusion

The presence of the phosphorylated form of Stat1 in mycobacterial infections after a long time from pathogen internalization has previously been demonstrated by us [4]. Excluding that the long-time p-tyr_701_Stat1 overexpression was caused by INFs release, we postulated that the JAK/STAT1 activity pathway could be due to bacterial replication in macrophage reservoir [4, 16]. The experimental results with cells-internalizing live *M. avium* showed two different moments of JAK/STAT1 pathway activation visible as increased expression of p-tyr_701_Stat1. The first peak of major expression of p-tyr_701_Stat1 was very evident at 24 hours especially in the control conditions where heat-killed *M. avium* and latex particles were used. Thus, we have highlighted a transient activity in the JAK/STAT1 pathway activation closely related to internalization events. In this work, we studied also JAK/STAT1 signalling pathway activation at 0–48 hours after internalization of both live and heat-killed *M. avium* in cultured human macrophages and its link with FcRs engagement. For this purpose, we investigated JAK/STAT1 pathway activity linked to receptor-mediated internalization events. The experiments conducted in parallel with macrophages internalizing live or heat-killed *M. avium* and latex particles (opsonized with different methods) demonstrate an important correlation between uptake engaging Fc*γ* membrane receptors and Stat1 phosphorylation. The JAK/STAT1 activity occurs shortly after internalization probably as a preventive defense mechanism; in fact, the expression levels of p-tyr_701_Stat1 are unrelated to the entity of materials internalized but related to Fc*γ*Rs engagement. The membrane receptors involvement in the internalization process could activate intracellular signaling pathways independent of the establishment of an active infection. Instead, a persistent intracellular p-tyr_701_Stat1 occurs only when an active infection is established. These data suggest that the JAK/STAT1 pathway is a new candidate for the development of new drugs against *M. avium* infection, but mechanisms that involve the transient Stat1 phosphorylation and are Fc related must be considered.

## Figures and Tables

**Table 1:** We correlate the Stat1/p-tyr_701_Stat1 densitometric value with the entity of internalized material in the presence or absence of Fc mAb IV.3 as Fc*γ*R inhibitor. Stat1 and p-tyr_701_Stat1 levels were normalized to the amount of actin and expressed as percentage of control value which was placed as 100%. The results are the mean of five different experiments. The control (CTR) for proteins' expression levels were represented by untreated macrophages. The correlation between internalization of Fc*γ*R and Stat1/p-tyr Stat1 pathway activity was determined at 24 hours after phagocytosis.

	Stat 1 (percent versus CTR)	p-tyr_701_Stat1 (percent versus CTR)	Number bacteria/cell
*M*. *avium* (AB)	200 ± 13.0	247 ± 12.0	12.2 ± 3.3
*M*. *avium* (AB) + Fc mAb IV.3	100 ± 12.4	113 ± 9.0	11.3 ± 2.2
			(95% versus serum AB)
CTR^∗^	100	100	—
HK *M. avium* (AB)	148 ± 7.2	658 ± 29.3	13.2 ± 3.5
HK *M*. *avium* (AB) + Fc mAb IV.3	112 ± 11.0	116 ± 5.0	5.0 ± 2.0
			(35% versus serum AB)

^∗^CTR (100%): human macrophages not exposed.
